# A novel extended flipped classroom model helps dental undergraduates grow into dentists

**DOI:** 10.1186/s12909-025-08525-5

**Published:** 2026-01-08

**Authors:** Ruikai Ba, Xinran Feng, Lixian Yuan, Juan Tong, Jinghao Ban, Meiling Wu, Bing Han, Xinyue Xu, Xinyu Qiu, Hao Guo, Kun Xuan

**Affiliations:** State Key Laboratory of Oral and Maxillofacial Reconstruction and Regeneration and National Clinical Research Center for Oral Diseases and Shaanxi Clinical Research Center for Oral Diseases, Department of Preventive Dentistry, School of Stomatology, The Fourth Military Medical University, Xi’an, 710032 China

## Abstract

**Background:**

The lack of the ability to flexibly and accurately apply theoretical knowledge learned in the classroom to clinical diagnosis and treatment has been one of the biggest barriers preventing dental undergraduates transition into competent dentists. The flipped classroom model and simulation training system supporting self-directed learning have been a significant shift in medical education. Literatures about these topics are abundant, however, there are few practical flipped-classroom models which could help dental undergraduates adapt to the status of dentists quickly. This study proposed a novel extended flipped classroom model (P-I-T-D-S Flipped Classroom Model) assisted by the simulation training system and verified its effectiveness.

**Methods:**

The scores of sophomores majored in the *Fundamentals of Stomatology* at Air Force Medical University participated in the novel extended flipped classroom model and those participated in the traditional lecture model are compared. Moreover, a total of 39 participants (2023 cohort) were stratified and randomly assigned into a pre-class group and a post-class group to explore better flipped classroom model. We descriptively analysed the quantitative data and thematically analysed the qualitative data.

**Results:**

The scores of the undergraduates enrolled in the P-I-T-D-S Flipped Classroom Model (2023) are significantly higher than those taught under the Traditional Lecture-Based Model (2022). Notably, the scores in the pre-class group raised significantly higher than those in the post-class group, which might be related to the active learning model. Additionally, the undergraduates in the P-I-T-D-S Flipped Classroom Model showed higher willingness to participate in and satisfaction of the class.

**Conclusion:**

The P-I-T-D-S flipped classroom medical education model, through active learning, can effectively stimulate the learning enthusiasm of dental students, thereby improving their learning efficiency of basic dental knowledge and clinical practice ability.

**Supplementary Information:**

The online version contains supplementary material available at 10.1186/s12909-025-08525-5.

## Introduction

 One of the biggest barriers preventing dental undergraduates grow into dentists is the lack of the ability to flexibly and accurately apply theoretical knowledge learned in the classroom to clinical diagnosis and treatment [1–4]. The main reasons include the following aspects: 1. Students lack comprehensive training in the analysis, diagnosis, and treatment plan design of complex cases using theoretical knowledge from subdisciplines such as prosthodontics, orthodontics, endodontics, and periodontology. 2. Dental undergraduates do not have sufficient communication training leading to psychological pressure which makes it more difficult for them to communicate with dental patients effectively and gain their trust [5, 6]. 3. The weak understanding of infection control, medical ethics and humanistic care are also huge obstacles for dental students to grow into qualified dentists [7, 8].

The core issue is to increase dental undergraduates’ preclinical practice training [9–11]. However, the fifth-year internship is limited especially since most patients are unwilling for interns to perform oral treatment on themselves. Therefore, we need to integrate the knowledge of oral diagnosis and treatment practice into the entire process of undergraduate education in dentistry, and provide sufficient simulation training, so that dental undergraduates can proficiently master the methods of communicating with and treating patients before entering clinical internships [12, 13]. Virtual reality simulators make it possible [14, 15]. Unidraw is one of the comprehensive dental simulators which combines virtual reality and haptic technology to provide students with immersive realistic diagnosis and treatment experience [16]. 3D treatment scenarios with Standardized oral disease patients are constructed in the dental situational clinical thinking training system. It can realistically simulate oral lesions and general situation, which enables students to connect key links throughout the diagnosis and treatment process in a virtual environment, and examines students’ abilities of theoretical knowledge mastery, history taking, imaging examination result interpretation, medical recording, clinical diagnosis and differential diagnosis, treatment plan formulation, etc. (Supplementary Video 1)

**Fig. 1:**
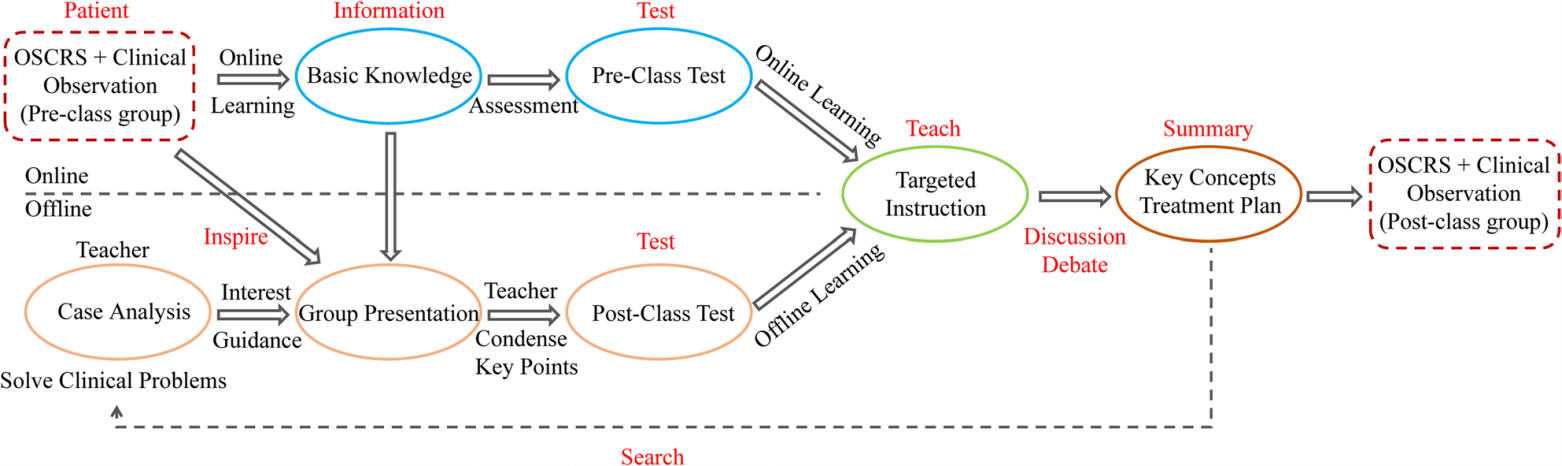
Study methodology flowchart

As a virtual simulation teaching assistance system, it also requires a scientific and reasonable mode to achieve the best educational effect [17–19]. The flipped (inverted) classroom has gained popularity in higher education in recent years [20–23]. Nevertheless, it is still unclear which is the best model for helping dental undergraduates grow into dentists. We propose a novel extended flipped classroom model called P-I-T-D-S flipped classroom model based on dental situational clinical thinking training system (Fig. 1). Each capital letter represents an implementing step for this model. ‘P’ represents patient and practice, which means case-based teaching and practice by dental situational clinical thinking training system. ‘I’ represents information and inspiration. Teachers assign self-learning tasks based on theoretical knowledge points and inspire students’ thinking on practical clinical problems during the clinical observation period. Students can finish pre-class preparation using information technology such as textbooks, MOOCs, PubMed, AI, etc., [24] combined with pre-class tasks. ‘T’ represents test and teach. Teachers conduct pre-class tests on pre-class tasks to identify students’ concentration problems during self-study, and provide targeted and focused teaching. ‘D’ represents discussion and debate. Teachers raise issues to allow students to design specific cases, organize group learning and discussion. Students prepare presentation materials with the information collected before class, and report on them in groups as one of the assessment criteria for daily grades. This enhances students’ enthusiasm and participation, deepens their understanding and practical application of knowledge points, and improves their teamwork and communication skills [20]. Moreover, teachers should encourage students to debate different design schemes for the same case, cultivate their clinical thinking and flexible adaptability in handling different cases, and stimulate their innovative thinking. ‘S’ represents summary and search. Teachers help students summarize the core of the course, cultivate students’ ability to summarize and generalize, and develop logical thinking. Based on the latest developments in the discipline, teachers and students propose appropriate treatment methods for real clinical cases, summarize the knowledge points involved in the cases, and summarize the key detailed treatment methods for this type of disease together. Teachers could introduce the latest scientific research progress, encourage students to engage in extended reading and learning after class, and cultivate their research abilities and interests. We apply this novel extended flipped classroom model in the *Fundamentals of Stomatology* for sophomores compared with traditional lecture-based mode to verify the improvement effect on learning. We also compared different using order of dental simulator and tried to make this novel mode more effective.

## Materials and methods

### Study participants and design

This study was conducted to evaluate the efficacy of the P-I-T-D-S flipped classroom model assisted by the Oral Scenario-Based Clinical Reasoning Training and Assessment System (OSCRS; Beijing Unidraw Virtual Reality Technology Institute Co., Ltd.) in enhancing pre-clinical competencies among second-year dental undergraduates at Air Force Medical University. We compared the scores of undergraduates (2023, experimental group (*N* = 39)) participated in the novel extended flipped classroom model and the undergraduates participated in the traditional lecture model (2022, control group (*N* = 38)). All of the participants assumed to have equivalent baseline levels and majored in the *Fundamentals of Stomatology*. Moreover, a total of 39 participants (2023) were stratified and randomly assigned into a pre-class group (OSCRS used before the class, *N* = 19) and a post-class group (OSCRS used after the class, *N* = 20) to explore better flipped classroom model. As we didn’t know whether the usage sequence of OSCRS training and clinical observation will influence the learning outcome, all participants provided informed consent for inclusion in this study. The Pre-class group received the OSCRS training and clinical observation module prior to the course, whereas the Post-class group received the same training after course completion. During the clinical observation component, students actively engaged in learning by observing real-time clinical procedures and asking questions, while instructors refrained from delivering direct knowledge or explanations during the session. This study was approved by the Ethics Committee of the school of Stomatology in the Air Force Medical University (KQ-YJ-2024-261).

### Eligibility and sampling

We conducted a census of all students enrolled in the target course across the 2022 (traditional lecture) and 2023 (P-I-T-D-S flipped) cohorts. Inclusion criteria were official enrollment during the study term and completion of core assessments (student satisfaction questionnaire and summative examinations/OSCE components). Exclusion criteria (pre-specified) were course withdrawal, repeated enrollment of the same course during the study window (duplicates removed at the student level), missing key outcomes, or attendance < 75% per university policy. Students meeting inclusion with partial missing covariates were retained for analyses in which data were complete (analysis-specific N reported).

### OSCRS training

The OSCRS platform employs virtual reality (VR), AI, and haptic feedback to simulate clinical scenarios featuring standardized patients with common oral pathologies, such as pulpitis, periodontal disease, and dental defects. Participants accessed a predefined pulp/periapical case (Case ID: 100136) via guest mode. The system workflow comprised AI-driven voice interaction for history-taking, utilizing a hierarchical question database (critical/general/non-essential) to evaluate query completeness and clinical logic. Haptic-enhanced oral examinations employed force-feedback devices (left hand: mouth mirror; right hand: probes/tweezers) to replicate tissue biomechanics within a 3D oral cavity model, with real-time visual cues highlighting pathologies such as caries or pulp exposure. Participants selected evidence-based auxiliary tests (e.g., periapical radiographs, vitality tests), with penalties for irrelevant or omitted choices. Diagnostic and treatment planning modules required submission of a primary diagnosis (supported by clinical findings), differential diagnoses, and clinically appropriate interventions. All procedural data were encrypted and synchronized to a secure backend database, enabling retrospective revisions.

### Effectiveness assessment

The *Fundamentals of Stomatology* is an innovative bridging course independently developed by our institution, aiming to integrate foundational clinical knowledge with practical clinical skills. The assessment framework of this course includes regular performance evaluations (60%) and examination scores (40%). The regular performance segment further comprises dental exam (30%) as well as dental cavity preparation (30%) (Supplementary Fig. 1). In the dental examination module, students participated in role-play exercises, alternating roles as clinicians and patients. The dental cavity preparation module utilized advanced simulation tooth-preparation models [25]. Upon course completion, students completed a structured questionnaire consisting of 19 objective items scored on a 1 ~ 5 Likert scale and one open-ended question. The survey evaluated multiple aspects including course content, instructional methods and their implementation, learning environment and support, self-regulated learning capabilities, clinical skills, professional competencies, and provided a section for open-ended feedback (Table 1, SupplementaryTable.1).

**Table 1:** Questionnaire of Students’ Learning Satisfaction

Categories	(1–5 Likert scale, where 1 = Strongly Disagree to 5 = Strongly Agree)
1	Do you perceive the course content as comprehensible?
2	Do you consider the course content beneficial for your disciplinary development?
3	How would you rate the level of engagement stimulated by the course content?
4	Do you actively engage in classroom pedagogical activities?
5	Is the instructional delivery aligned with the predetermined curriculum schedule?
6	Are you satisfied with the pedagogical methodologies employed in this course?
7	How would you evaluate the adequacy of instructional materials utilized in this course?
8	Do you consider the assessment framework of this course to be appropriate?
9	Do you perceive the in-class learning environment as conducive to academic engagement?
10	How would you assess the adequacy of extracurricular learning support systems?
11	Do you find the self-regulated learning strategies implemented in this course effective?
12	Are you satisfied with the instructional design architecture of this course?
13	How would you rate the quality of teacher-student interaction in this course?
14	Has this course enhanced your academic self-efficacy and learning autonomy?
15	Do you believe this course has enabled you to acquire clinical oral examination competencies?
16	Have you developed diagnostic proficiency for oral pathologies through this course?
17	Have you attained treatment plan formulation skills through this instructional program?
18	Has this course improved your capacity for independent scholarship?
19	Do you perceive enhanced critical thinking skills in clinical decision-making through this course?
20	Recommendations for course improvement: (Qualitative response)

*Reliability/validity note*: α 0.93; ω 0.94; dimension-level α 0.82–0.89; I-CVI 0.83–1.00; S-CVI/Ave 0.94; inter-rater ICC(2,1): 0.84 (Dental Exam), 0.81 (Cavity Preparation); weighted κ 0.76–0.85.

### Questionnaire validation and rater calibration

The 19-item Likert questionnaire underwent content validation by a six-member expert panel (I-CVI 0.83–1.00; S-CVI/Ave 0.94). Internal consistency was high (Cronbach’s α 0.93; McDonald’s ω 0.94; dimension-level α 0.82–0.89). Two faculty evaluators were trained with rubric/anchor exemplars and achieved ≥ 90% agreement on a 15% practice set; raters were blinded during scoring. Inter-rater reliability in the study set was summarized by ICC (2, 1) and weighted κ.

### Qualitative thematic analysis

We thematically analyzed open-ended comments from the 2022 and 2023 cohorts. Two trained coders independently performed inductive, semantic-level open coding in [NVivo/Atlas.ti/Excel], iteratively developed a codebook, and reconciled discrepancies by consensus; a third reviewer was available for arbitration. Inter-coder reliability on a random 20% subsample was substantial (Cohen’s κ = 0.78, 95% CI 0.70–0.86; raw agreement 87%). Codes were clustered into higher-order categories to generate themes and subthemes via constant comparison. De-identified representative quotations (lightly edited for readability without altering meaning) were compiled. For example, we edited the original expression “Um, I feel like this flipped classroom is kind of more helpful for us than, you know, the traditional lecture.” to “I feel that this flipped classroom is more helpful for us than the traditional lecture.” Two word-clouds remain in the main text as descriptive visuals and were not used to derive themes. A thematic map and a theme summary are presented in the main text as Fig. 6; Table 6, respectively.

### Statistical analysis

All analyses were conducted in *R* (version 4.2). Continuous and Likert-type variables were summarised with medians and inter-quartile ranges, whereas categorical data were reported as proportions. Between group differences in continuous or ordinal outcomes were evaluated with the Wilcoxon rank-sum test after the Shapiro–Wilk test indicated departures from normality. Item-level Likert scores were compared with two complementary non-parametric procedures: (i) the Mann–Whitney U test to assess shifts across the full ordinal distribution and (ii) Pearson’s χ² test applied to a dichotomised “satisfied” threshold (scores 4–5 versus 1–3) to generate intuitive effect sizes for graphical overlays. False-discovery control across multiple questionnaire items was achieved with the Benjamini–Hochberg adjustment, accepting an adjusted *p* < 0.05 as statistically significant. For multivariate visualisation, item scores were rescaled to the 0–1 interval relative to the satisfaction cut-off and plotted as radar polygons to depict changes across pedagogical dimensions. Open-ended responses were summarized using the thematic procedures above. Theme frequencies were presented descriptively (n, %), where n denotes the number of unique open-ended comments in which a given theme was identified (presence/absence per comment); % uses the total number of open-ended comments in the relevant set (cohort-specific or pooled). Because themes can co-occur within the same comment, percentages may sum to > 100%. The two word clouds in the main text are descriptive only and were not used for theme generation. Reliability/validity indices reported include Cronbach’s α, McDonald’s ω, I-CVI/S-CVI(Ave), and inter-rater ICC(2,1) with checklist-level weighted κ (two-sided 95% CIs where applicable). Power benchmarking indicated that detecting d = 0.35 at α = 0.05 and 80% power requires ~ 130 per group; the achieved sample exceeded this threshold. All graphics were produced with the ggplot2, ggradar, and patchwork libraries.

## Results

### Comparison of student performance between the traditional lecture-based model (2022) and the P-I-T-D-S flipped classroom model (2023)

A total of two cohorts were compared: students taught under the Traditional Lecture-Based Model (2022) and those enrolled in the P-I-T-D-S Flipped Classroom Model (2023). All results are reported as median [interquartile range], and comparisons were conducted using the Wilcoxon rank-sum test.

As shown in Table 2, students in the Flipped classroom group achieved significantly higher scores in several key assessments. The Dental Exam score in the flipped classroom group was 90.0 [89.0–100.0], compared to 88.0 [83.0–93.0] in the traditional group (*p* = 0.003). Similarly, the Test score significantly improved from 76.0 [76.0–82.0] in the traditional model to 84.0 [79.0–89.5] in the flipped model (*p* < 0.001). The Total Score also increased significantly (*p* < 0.001), rising from 76.3 [73.9–78.7] to 81.9 [76.2–87.2]. All pre-specified eligibility criteria and exclusions were applied; the final analytic dataset comprised all eligible students with complete data for each analysis.

**Table 2:** Statistical Comparison Between the Traditional Lecture-based Model (2022) and the P-I-T-D-S Flipped Classroom Model (2023). Values are expressed as median [IQR]. Comparisons were performed using the Wilcoxon rank-sum test

Variable	Traditional Lecture-based Model (Median [IQR])	*P*-I-T-D-S Flipped Classroom Model (Median [IQR])	*p*-value
Dental Cavity Preparation	60.0 [60.0–68.8]	65.0 [52.5–80.0]	0.30600
Dental Exam	88.0 [83.0–93.0]	90.0 [89.0–100.0]	0.00316
Test	76.0 [76.0–82.0]	84.0 [79.0–89.5]	< 0.001
Total Score	76.3 [73.9–78.7]	81.9 [76.2–87.2]	< 0.001

Although the Dental Cavity Preparation score was higher in the flipped group (65.0 [52.5–80.0]) than in the traditional group (60.0 [60.0–68.8]), this difference did not reach statistical significance (*p* = 0.306).

**Fig. 2:**
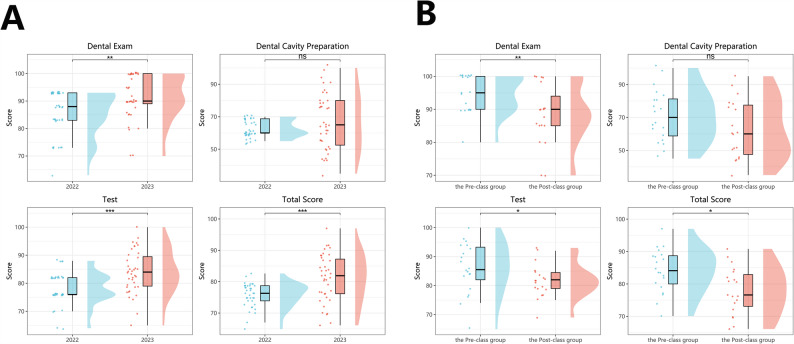
Comparison of student performance between the traditional lecture-based model and the P-I-T-D-S flipped classroom model as well as between the Pre-class group and Post-class group under the P-I-T-D-S flipped classroom model. **A** The box plots represent median and interquartile ranges for each evaluation metric, including Dental Cavity Preparation, Dental Exam, Test, and Total Score. Data from the 2022 cohort (traditional model) and 2023 cohort (P-I-T-D-S model) are shown. Statistically significant differences were observed in the Dental Exam, Test, and Total Score (*p* < 0.01). **B** Box plots represent the median and interquartile range of scores for each assessment item, including Dental Cavity Preparation, Dental Exam, Test, and Total Score. Statistically significant improvements were observed in the Dental Exam, Test, and Total Score following the full implementation of the instructional sequence (*p* < 0.05, Wilcoxon rank-sum test)

These findings are visually supported by Fig. 2A, which illustrates consistent score improvements across all categories under the P-I-T-D-S model, highlighting its effectiveness in enhancing student academic performance in clinical dentistry.

### Comparison of student performance between the pre-class group and the post-class group within the P-I-T-D-S flipped classroom model (2023)

To evaluate the effect of OSCRS training timing within the P-I-T-D-S flipped classroom model, student performance was compared between the Pre-class group and the Post-class group, both from the 2023 cohort. All values are presented as median [interquartile range], and comparisons were conducted using the Wilcoxon rank-sum test.

As summarized in Table 3, the Pre-class group demonstrated significantly higher academic performance across multiple evaluation metrics. The Dental Exam score increased from 90.0 [85.0–94.0] in the Post-class group to 95.0 [90.0–100.0] in the Pre-class group (*p* = 0.00871). Similarly, the Test score improved from 82.0 [79.0–84.5] to 85.5 [82.0–93.2] (*p* = 0.04880). The Total Score, reflecting overall student performance, rose from 76.6 [73.1–82.9] to 84.1 [80.0–88.7], also showing statistical significance (*p* = 0.0101).

**Table 3:** Statistical Comparison Between Pre-Class and Post-Class Groups (2023). Values are expressed as median [IQR]. Comparisons between groups were conducted using the Wilcoxon rank-sum test

Variable	Pre-Class Group (Median [IQR])	Post-Class Group (Median [IQR])	*p*-value
Dental Cavity Preparation	70.0 [58.8–81.2]	60.0 [47.5–77.5]	0.11400
Dental Exam	95.0 [90.0–100.0]	90.0 [85.0–94.0]	0.00871
Test	85.5 [82.0–93.2]	82.0 [79.0–84.5]	0.04880
Total Score	84.1 [80.0–88.7]	76.6 [73.1–82.9]	0.01010

Although the Dental Cavity Preparation score was higher in the Pre-class **g**roup (70.0 [58.8–81.2]) than in the Post-class group (60.0 [47.5–77.5]), this difference did not reach statistical significance (*p* = 0.114).(Fig. 2B).

These results suggest that the structured deployment of OSCRS training within the P-I-T-D-S model may yield cumulative benefits to student learning outcomes. The OSCRS training and clinical observation module prior to the course should be a better model.

### Results of the student satisfaction questionnaire

#### Student-reported satisfaction

A comparison of student satisfaction rates between the traditional lecture-based model (2022) and the P-I-T-D-S flipped classroom model (2023) revealed a statistically significant improvement in favor of the flipped classroom. Among the 19 survey items, five questions showed significant differences based on chi-square tests after aggregating satisfaction scores of 4–5: Q17 (χ² *p* = 0.0032), Q9 (*p* = 0.0104), Q19 (*p* = 0.0234), Q11 (*p* = 0.0440), and Q18 (*p* = 0.0462). The largest improvement was observed for Q17 (mean score: 3.97 in 2022 vs. 4.67 in 2023), with a delta of 0.69.

**Fig. 3:**
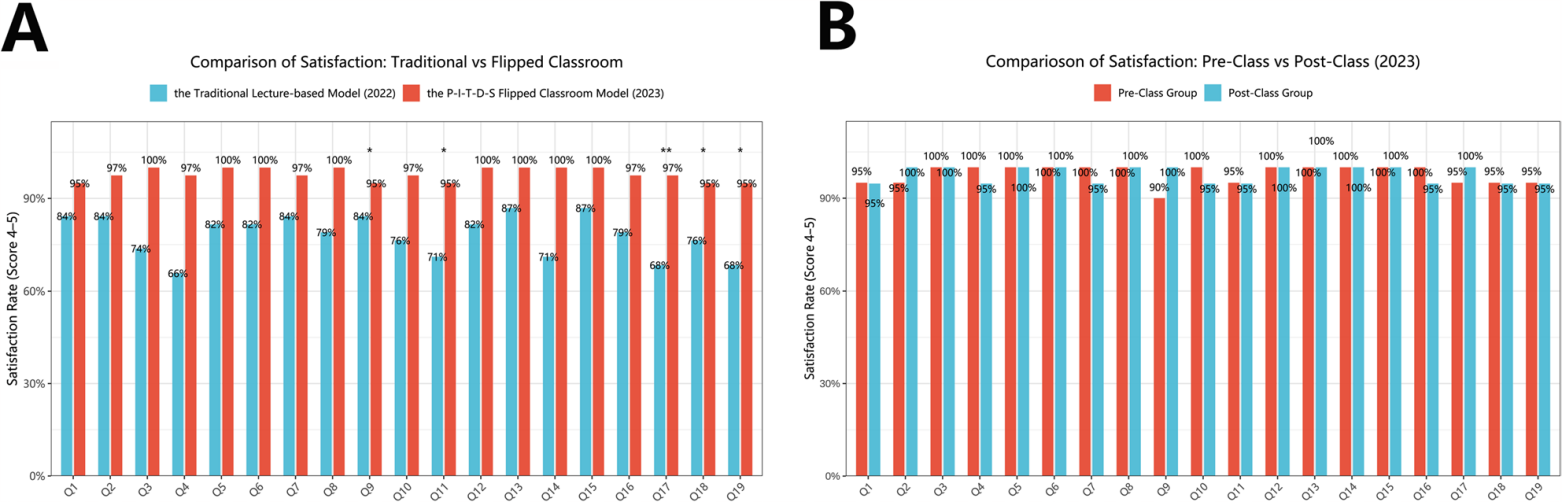
Comparison of student satisfaction rates (scores of 4–5) between 2022 and 2023 cohorts as well as between Pre-class group and Post-class group in 2023. **A** Comparison of student satisfaction rates (scores of 4–5) between the traditional lecture-based cohort (2022) and the P-I-T-D-S flipped classroom cohort (2023). Each bar represents the percentage of students assigning a satisfaction score of 4 or 5 on a 5-point Likert scale for each questionnaire item (Q1–Q19). Red bars denote the flipped classroom group and blue bars denote the traditional group. Asterisks above the bars indicate statistical significance based on chi-square tests: **p < 0.001, *p < 0.01, *p* < 0.05, ns = not significant. Questions are sorted by item number; satisfaction was consistently higher in the flipped classroom group across most items. **B** Comparison of student satisfaction rates (scores of 4–5) between the Pre-class group and Post-class group within the 2023 P-I-T-D-S flipped classroom cohort. Bars represent the proportion of students rating each item with a satisfaction score of 4 or 5 on a 5-point Likert scale (Q1–Q19). Red bars represent the Pre-class group and blue bars represent the Post-class group. No statistically significant differences were detected by chi-square tests across all items (ns = not significant). An asterisk (*) denotes marginal significance in Wilcoxon rank-sum test (Q7, *p* < 0.05), indicating higher satisfaction in the Pre-class group

Additionally, Wilcoxon rank-sum tests indicated significant improvements in 18 out of 19 items (*p* < 0.05), supporting the overall enhancement in satisfaction following the implementation of the flipped classroom. Several questions (e.g., Q3, Q4, Q6, Q16) exhibited notable mean score differences exceeding 0.6 in favor of the 2023 cohort. These results highlight a consistent and broad-based increase in perceived satisfaction among students exposed to the P-I-T-D-S teaching model. (Table 4; Fig. 3A).

**Table 4:** Comparison of student satisfaction rates (scores of 4–5) between the traditional lecture-based model (2022) and the P-I-T-D-S flipped classroom mode (2023)

Question	chisq_p	u_p	the Traditional Lecture-based Model (2022)	the *P*-I-T-D-S Flipped Classroom Model (2023)	delta
Q17	0.003244185	0.000498	3.973684211	4.666666667	0.692982456
Q9	0.010384502	0.00175	4.289473684	4.769230769	0.479757085
Q19	0.023391189	0.00135	4.026315789	4.692307692	0.665991903
Q11	0.044010293	0.00309	4.078947368	4.692307692	0.613360324
Q18	0.0462163	0.00236	4.131578947	4.717948718	0.586369771
Q1	NS	0.0236	4.289473684	4.641025641	0.351551957
Q10	NS	0.00313	4.210526316	4.769230769	0.558704453
Q12	NS	0.000537	4.289473684	4.871794872	0.582321188
Q13	NS	0.00136	4.368421053	4.871794872	0.503373819
Q14	NS	0.0016	4.157894737	4.794871795	0.636977058
Q15	NS	0.000868	4.342105263	4.820512821	0.478407557
Q16	NS	0.000217	4.131578947	4.769230769	0.637651822
Q2	NS	0.000437	4.315789474	4.846153846	0.530364372
Q3	NS	0.000082	4.157894737	4.871794872	0.713900135
Q4	NS	0.000806	4.052631579	4.743589744	0.690958165
Q5	NS	0.00225	4.342105263	4.871794872	0.529689609
Q6	NS	0.0000942	4.263157895	4.897435897	0.634278003
Q7	NS	0.0091	4.236842105	4.743589744	0.506747638
Q8	NS	0.000319	4.315789474	4.923076923	0.607287449

Within the 2023 P-I-T-D-S flipped classroom cohort, a subgroup analysis comparing the Pre-class group and the Post-class group showed no statistically significant differences in satisfaction scores based on chi-square tests across any of the 19 survey items (*p* > 0.05 for all items). Similarly, Wilcoxon rank-sum tests revealed no significant differences for the majority of questions, with the exception of Q7 (*p* = 0.048), where the Pre-class group reported a higher satisfaction mean (4.90 vs. 4.58), yielding a delta of + 0.32.

Despite the overall lack of statistically significant differences, the Pre-class group exhibited consistently higher mean scores across most items. For example, Q11 (4.80 vs. 4.58), Q12 (4.95 vs. 4.79), Q16 (4.90 vs. 4.63), and Q18 (4.80 vs. 4.63) demonstrated moderate deltas ranging from + 0.16 to + 0.27. These trends suggest a potentially more favorable reception to the flipped classroom content when students were exposed to OSCRS training prior to in-class delivery, warranting further investigation in larger samples or across multiple semesters (Table 5; Fig. 3B).

**Table 5:** Differences Between the Post-Class and Pre-Class groups under the P-I-T-D-S flipped classroom model (2023)

question	chisq_p	u_p	Post-Class Group	Pre-Class Group	delta
Q1	NS	0.931	4.631578947	4.65	0.018421053
Q10	NS	0.368	4.684210526	4.85	0.165789474
Q11	NS	0.15	4.578947368	4.8	0.221052632
Q12	NS	0.146	4.789473684	4.95	0.160526316
Q13	NS	0.611	4.842105263	4.9	0.057894737
Q14	NS	0.399	4.736842105	4.85	0.113157895
Q15	NS	0.197	4.736842105	4.9	0.163157895
Q16	NS	0.0965	4.631578947	4.9	0.268421053
Q17	NS	0.747	4.684210526	4.65	-0.034210526
Q18	NS	0.259	4.631578947	4.8	0.168421053
Q19	NS	0.45	4.631578947	4.75	0.118421053
Q2	NS	0.663	4.842105263	4.85	0.007894737
Q3	NS	0.611	4.842105263	4.9	0.057894737
Q4	NS	0.863	4.736842105	4.75	0.013157895
Q5	NS	0.611	4.842105263	4.9	0.057894737
Q6	NS	0.979	4.894736842	4.9	0.005263158
Q7	NS	0.0482	4.578947368	4.9	0.321052632
Q8	NS	0.543	4.894736842	4.95	0.055263158
Q9	NS	0.964	4.842105263	4.7	-0.142105263

#### Radar comparison of key teaching dimensions across students groups

Aggregating the 19 questionnaire items into five pedagogical dimensions showed that student satisfaction rose markedly after implementation of the P-I-T-D-S flipped-classroom model: overall mean Likert score increased from 4.25 ± 0.31 in the 2022 traditional cohort to 4.78 ± 0.22 in the 2023 flipped cohort (Δ + 0.53, *p* < 0.001); the greatest gain was in Instructional Implementation (4.19 → 4.81, Δ + 0.62, *p* < 0.001), followed by Clinical Competence Acquisition (4.04 → 4.62, Δ + 0.58, *p* < 0.001), Self-Regulated Learning Support (Δ + 0.49, *p* < 0.01) and Course Content & Structure (Δ + 0.45, *p* < 0.01), while Teacher–Student Interaction remained highest in both cohorts (4.70 → 4.93, Δ + 0.23, *p* = 0.012).

**Fig. 4:**
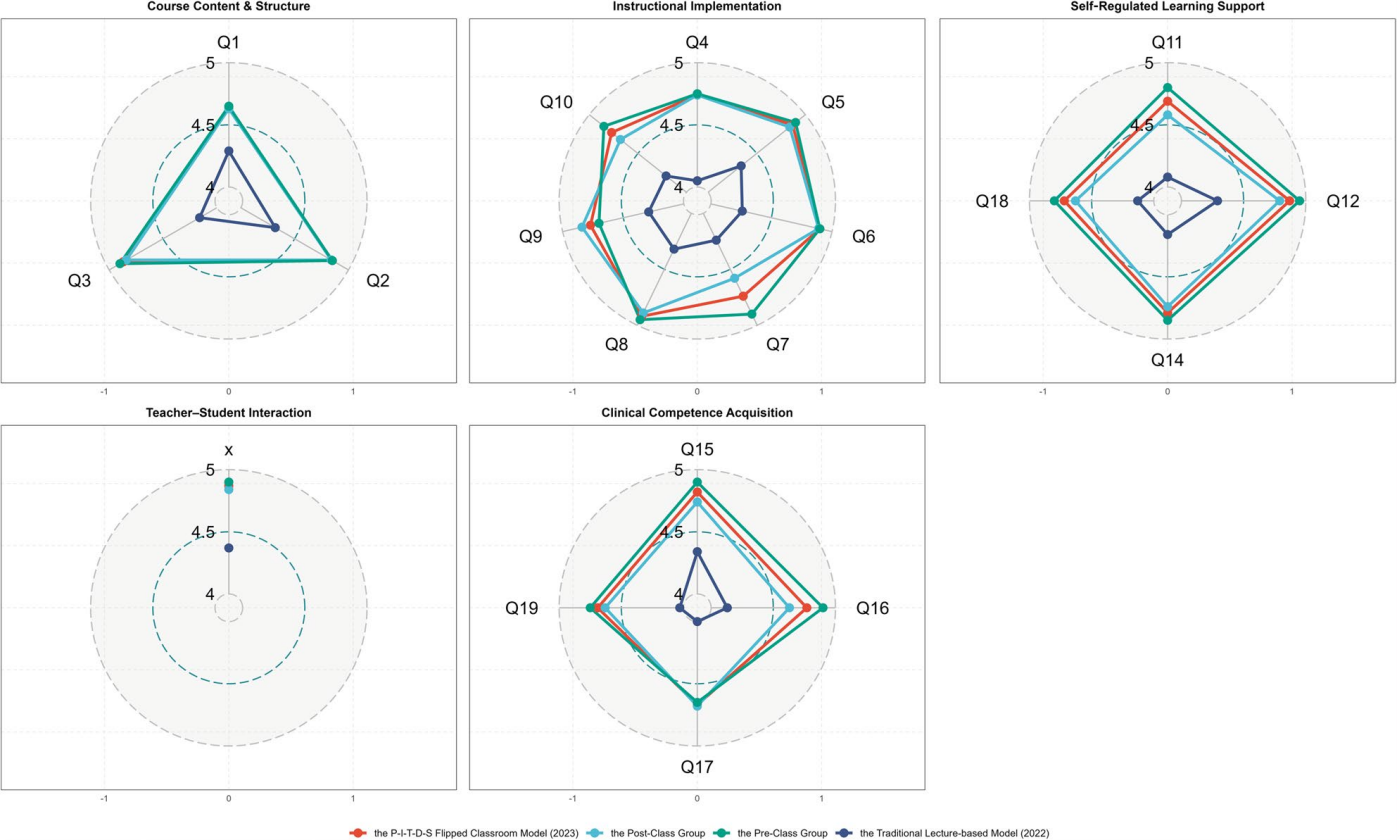
Radar Comparison of Categorized Teaching Dimensions Across Student Groups

Within the 2023 cohort, no statistically significant differences emerged between the Pre-class group and the Post-class group across any of the five teaching dimensions (all *p* > 0.05), indicating that the flipped classroom design delivered uniformly high satisfaction regardless of the timing of OSCRS training (Fig. 4).

Radar plot of mean satisfaction (scaled 4–5) across five dimensions for the Traditional Lecture-based Model (2022, blue), the P-I-T-D-S Flipped Classroom Model (2023, red), the Pre-class group (orange), and the Post-class group (green). Concentric rings correspond to Likert 4, 4.5, and 5, and a larger radial polygon denotes higher satisfaction. A single legend appears at the bottom to avoid redundancy across the five panels.

#### Instrument validation and rater calibration

Instrument validation and rater calibration. The questionnaire showed strong internal consistency (α 0.93; ω 0.94; dimension-level α 0.82–0.89) and high content validity (I-CVI 0.83–1.00; S-CVI/Ave 0.94). Inter-rater reliability was ICC(2,1) 0.84 for the Dental Exam and 0.81 for Cavity Preparation (checklist-level weighted κ 0.76–0.85). These metrics confirm an adequately validated instrument and calibrated evaluators.

#### Qualitative findings and cross-year contrast

**Fig. 5:**
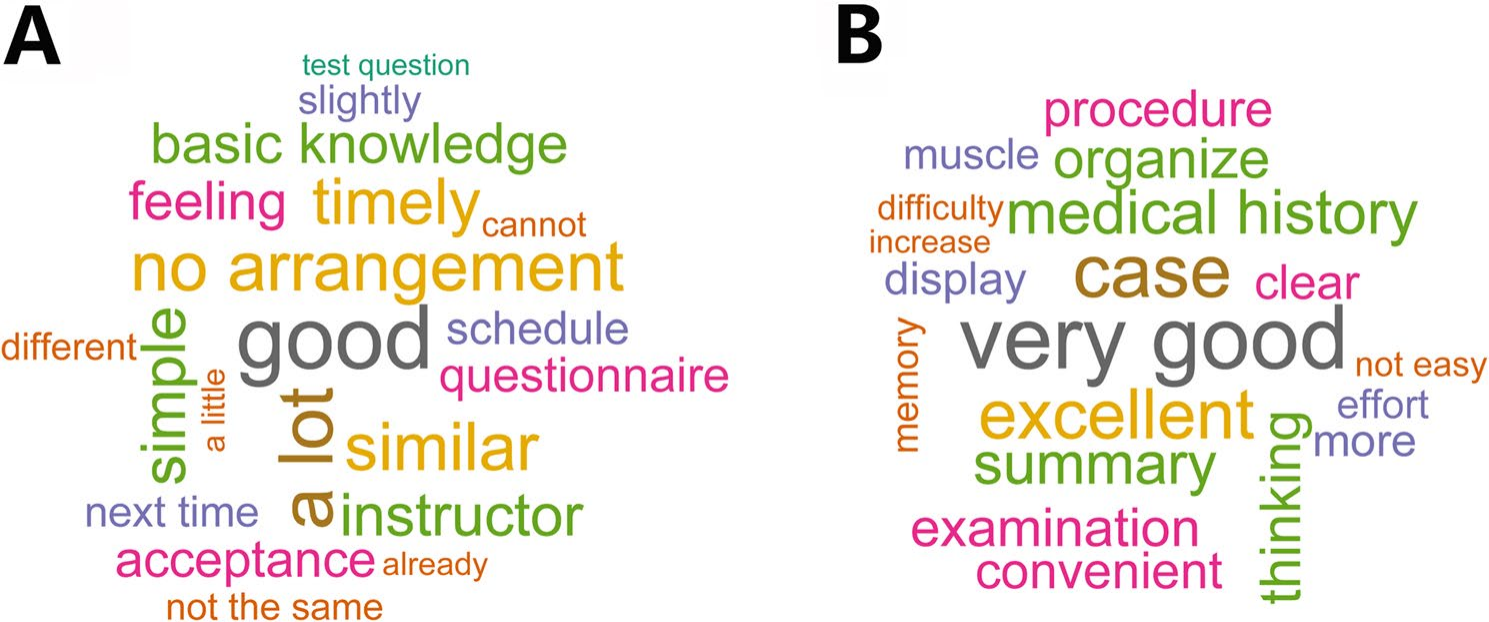
Word cloud visualization of students’ suggestions from open-ended questionnaire responses in 2022 and 2023. The figure displays the relative frequency of terms mentioned by students. Larger words represent higher frequencies. The 2023 responses (**B**) show a more positive and cohesive focus compared to 2022 (**A**), reflecting increased satisfaction and engagement following instructional enhancements

Descriptive signals from the two word clouds: The 2022 cloud (in Fig. 5A) emphasizes logistics/clarity and low difficulty (e.g., arrangement/timely, clear, basic knowledge, quiz/questionnaire, simple/easy). In 2023 (in Fig. 5B), tokens shift toward pedagogy and skills (e.g., cases, practice, method, thinking, demonstration/examination/step-by-step operation), with positive valence (very good, convenient) and “not easy,” suggesting improved difficulty calibration.

**Fig. 6:**
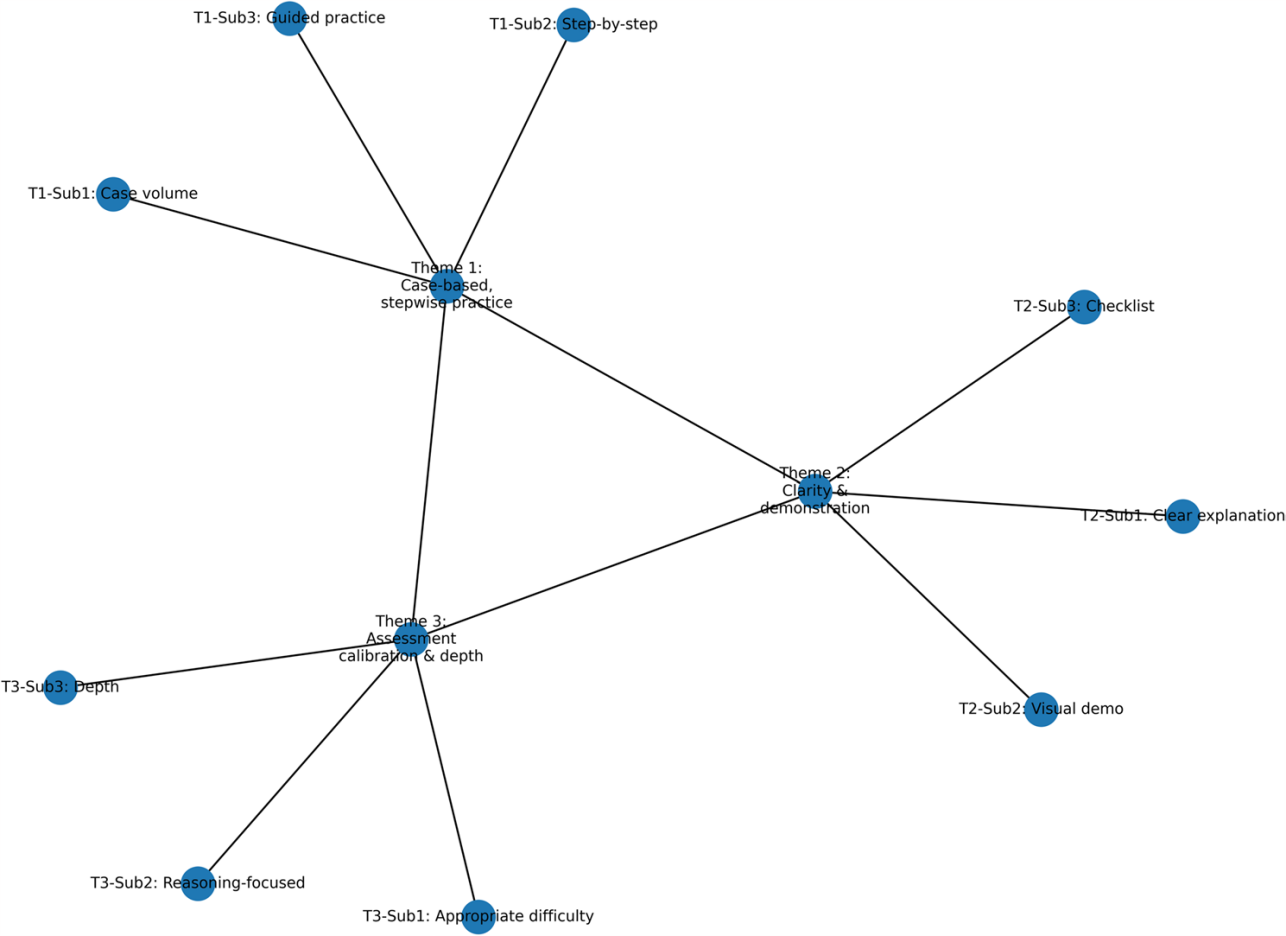
Thematic map of student feedback. Nodes represent themes and subthemes; connections indicate conceptual relatedness. This figure summarizes how case-based stepwise practice, clarity with demonstration, and assessment calibration interrelate across cohorts

Themes from coding (co-occurrence allowed): Three cross-cutting themes emerged— (1) case-based, stepwise practice (~ 42%); (2) clarity & demonstration (~ 36%); and (3) assessment calibration & depth (~ 28%). These themes triangulate with the 2023 quantitative gains. The thematic map and the theme summary are shown in Fig. 6; Table 6, respectively.

**Table 6:** Summary of qualitative themes derived from open-ended comments across cohorts. The table lists themes, concise definitions, illustrative subthemes, and de-identified representative quotations, with descriptive frequencies (n, %). n denotes the number of unique comments in which the theme was identified; % is n divided by the total number of open-ended comments in the relevant set. Because themes can co-occur, percentages may sum to 100%

Theme	Definition (1–2 sentences)	Subthemes	Representative quotes (IDs)	*n*	Percent
Theme 1: Case-based, stepwise practice	Requests for more cases, standardized procedures, and guided hands-on practice.	Case volume; Step-by-step procedure; Guided practice	“Add more real cases and detailed steps.” (ID-xx); “Stepwise demos help link procedures with reasoning.” (ID-yy)	—	42%
Theme 2: Clarity & demonstration	Value of clear explanations and visual/step demonstrations aligned with reasoning.	Clear explanation; Visual demonstration; Checklist alignment	“Demonstrations make abstract content clear.” (ID-zz)	—	36%
Theme 3: Assessment calibration & depth	Shift from easy/simple to appropriately challenging, application-oriented assessment.	Appropriate difficulty; Reasoning-focused quiz; Depth of content	“Quizzes should test reasoning, not recall.” (ID-aa)	—	28%

## Discussion

### Advantages of the P-I-T-D-S flipped classroom model

Increasing clinical training is a necessary guarantee for improving the clinical skills of dentists [26, 27]. How to improve the clinical practice level of dental students while ensuring patient efficacy has always been a question that dental educators are considering [28, 29]. In the previous clinical training mode, students were trained to operate treatment in plaster teeth, detached teeth, and simulated head models. After passing the examination by the clinical instructors, patients underwent clinical treatment by dental interns under the supervision of the instructors. This clinical internship method has many problems, such as affecting clinical work efficiency, hidden dangers in the early treatment effect of interns, and patients’ resistance to interns’ treatment. The emergence of virtual simulation clinical training systems provides the possibility to solve these problems [30, 31]. How to integrate virtual simulation clinical training system into undergraduate education and construct a teaching plan that combines virtual and real elements to effectively enhance the clinical practice ability as well as diagnosis and treatment level of dental undergraduate students is a question that every dental educator needs to consider [32–34]. The author integrates existing flipped classroom models with Oral Scenario-Based Clinical Reasoning Training and Assessment System and proposes a P-I-T-D-S flipped classroom medical education universal model based on oral clinical teaching practice and related research results. By introducing real clinical cases, utilizing information technology teaching methods and AI virtual simulation training system (Unidraw) to stimulate students’ interest in learning, they can independently collect data, prepare basic knowledge before class, conduct clinical reception process and treatment practical training. Through classroom reports and case analysis discussions and debates, they can cultivate clinical diagnosis and treatment thinking and personalized treatment plan design thinking, enhance students’ teamwork ability and comprehensive analysis and solution ability for common and complex oral diseases. Finally, by summarizing and condensing the key points of the course to deepen the memory of key content, while also exploring new research hotspots and thinking about innovative research directions, it lays a foundation for future graduate studies. Dental undergraduates who have undergone a comprehensive combination of virtual and real clinical teaching could effectively reduce the trial and error costs during clinical internships to treat real patients. The P-I-T-D-S flipped classroom medical education model is not only suitable for dental education, but can also be expanded into a standardized model for clinical medical education in the future, with good universality.

### The relationship of active learning based P-I-T-D-S model with course experience, engagement, generic skills, and examination performance

The P-I-T-D-S flipped classroom medical education model can effectively improve the clinical teaching effectiveness of undergraduate dental students because its core concept is active learning. Arnaldo Perez et al. found that active learning can effectively improve the teaching effectiveness of dental undergraduates [35]. The focus of this project is the relationship of active learning based P-I-T-D-S model with course experience, engagement, generic skills, and examination performance. The research results show that compared with undergraduate students of the same level in 2022 who adopt the traditional lecture teaching mode, the mean score of students in P-I-T-D-S model is significantly higher than that of the students in traditional lecture model. ‘High-yield’ learning techniques in the study time are preferred by students driven by a ‘cost–benefit’ analysis which balances assessment demands against efficient time management [36]. OSCRS training system in P-I-T-D-S model provides students with time-saving and most clinically relevant real training scenarios, meets their needs for early clinical exposure, and stimulate their interest in exploration, thereby enabling them to improve their learning efficiency and effectiveness through self-directed learning and research. Higher mean scores and satisfaction rates shows the advantages of P-I-T-D-S model for improving the course experience, engagement, generic skills, and examination performance. Interestingly, we found that the scores in the Pre-class group were significantly higher compared to the Post-class group on both the test and the Dental Exam, which indicated that OSCRS training before class could stimulate students’ learning enthusiasm and enhance their learning effect and clinical practice ability.

The qualitative shift from logistics/clarity (2022) toward case-based, stepwise practice (2023) suggests that calibrated difficulty and explicit demonstrations that link procedures to reasoning are pivotal mechanisms for improving learning. Accordingly, increasing the volume/complexity of case vignettes and live/recorded demonstrations, standardizing step-by-step checklists for common examinations/procedures with guided practice, and recalibrating formative quizzes toward application and reasoning may sustain the observed gains.

### Strengths and limitations

The limited number of students leads to the need for further improvement in the scale of the experiment. But this experiment involved a wider range of student groups with different starting points and learning levels, such as five-year and eight-year programs. The corroboration between quantitative and qualitative data enhances the validity of the results. The mixed methods of this study also provide the possibility for enhancing the completeness, comprehensibility, and applicability of research results by obtaining quantitative information from larger sample sizes in the future, while qualitative data allows us to gain a more detailed understanding of the reasons why students have a special perspective on active learning. Due to the fact that we didn’t draw the dental cavity preparation simulation training module into our research, there was no significant difference between the experimental group (2023) and the control group (2022). In the future, we will compare the impact of the use of demand side systems on the teaching effectiveness of procedures such as cavity preparation and full crown teeth preparation in oral professional courses. In the next step of our research, we will further expand the sample size and application scope, and attempt to apply the P-I-T-D-S flipped classroom medical education model in clinical medical education, and study its application effect and possible problems. Through continuous improvement and enhancement, we aim to improve the P-I-T-D-S flipped classroom medical education model in order to achieve wider application and better teaching effectiveness, which will help more dental and clinical students grow smoothly into effective dentists and clinical doctors.

## Conclusions

The P-I-T-D-S flipped classroom medical education model, through active learning, can effectively stimulate the learning enthusiasm of dental students, thereby improving their learning efficiency of basic dental knowledge and clinical practice ability.

## Supplementary Information

Supplementary Material 1.

Supplementary Material 2.

Supplementary Material 3.

## Data Availability

The datasets generated during and analyzed during the current study are available from the corresponding author.
