# Supplementary material for: A novel extended flipped classroom model helps dental undergraduates grow into dentists
Source: BMC Med Educ. 2026 Jan 8;26:200. doi: 10.1186/s12909-025-08525-5 (PMC12870994; doi:10.1186/s12909-025-08525-5)
Supplement: Supplementary file 2 — Supplementary Material 2. [file 12909_2025_8525_MOESM2_ESM.docx]

# Questionnaire of Students’ Learning Satisfaction

| **Categories** | **(1-5 Likert scale, where 1=Strongly Disagree to 5=Strongly Agree)** | **The score** |
| --- | --- | --- |
| **1** | Do you perceive the course content as comprehensible? |  |
| **2** | Do you consider the course content beneficial for your disciplinary development? |  |
| **3** | How would you rate the level of engagement stimulated by the course content? |  |
| **4** | Do you actively engage in classroom pedagogical activities? |  |
| **5** | Is the instructional delivery aligned with the predetermined curriculum schedule? |  |
| **6** | Are you satisfied with the pedagogical methodologies employed in this course? |  |
| **7** | How would you evaluate the adequacy of instructional materials utilized in this course? |  |
| **8** | Do you consider the assessment framework of this course to be appropriate? |  |
| **9** | Do you perceive the in-class learning environment as conducive to academic engagement? |  |
| **10** | How would you assess the adequacy of extracurricular learning support systems? |  |
| **11** | Do you find the self-regulated learning strategies implemented in this course effective? |  |
| **12** | Are you satisfied with the instructional design architecture of this course? |  |
| **13** | How would you rate the quality of teacher-student interaction in this course? |  |
| **14** | Has this course enhanced your academic self-efficacy and learning autonomy? |  |
| **15** | Do you believe this course has enabled you to acquire clinical oral examination competencies? |  |
| **16** | Have you developed diagnostic proficiency for oral pathologies through this course? |  |
| **17** | Have you attained treatment plan formulation skills through this instructional program? |  |
| **18** | Has this course improved your capacity for independent scholarship? |  |
| **19** | Do you perceive enhanced critical thinking skills in clinical decision-making through this course? |  |
| **20** | Recommendations for course improvement: (Qualitative response) |  |

**Pedagogy:** The teaching methods and strategies used in this course (how the teacher teaches and organizes activities).

**Self-efficacy:** Your confidence that you can successfully complete the learning tasks in this course.

**Disciplinary development:** Your progress and growth in your professional field (dentistry), including knowledge, skills and attitudes.

**Flipped classroom:** A teaching model where you learn the main content before class and use class time mainly for discussion and practice.

**Pre-class learning:** Activities you do before class, such as watching videos or reading materials.

**In-class learning:** Activities during class, such as group discussion, case analysis and practice.

**Post-class learning:** Activities after class, such as homework, review and reflection.
