# Supplementary figures and images for: A novel extended flipped classroom model helps dental undergraduates grow into dentists
Source: BMC Med Educ. 2026 Jan 8;26:200. doi: 10.1186/s12909-025-08525-5 (PMC12870994; doi:10.1186/s12909-025-08525-5)

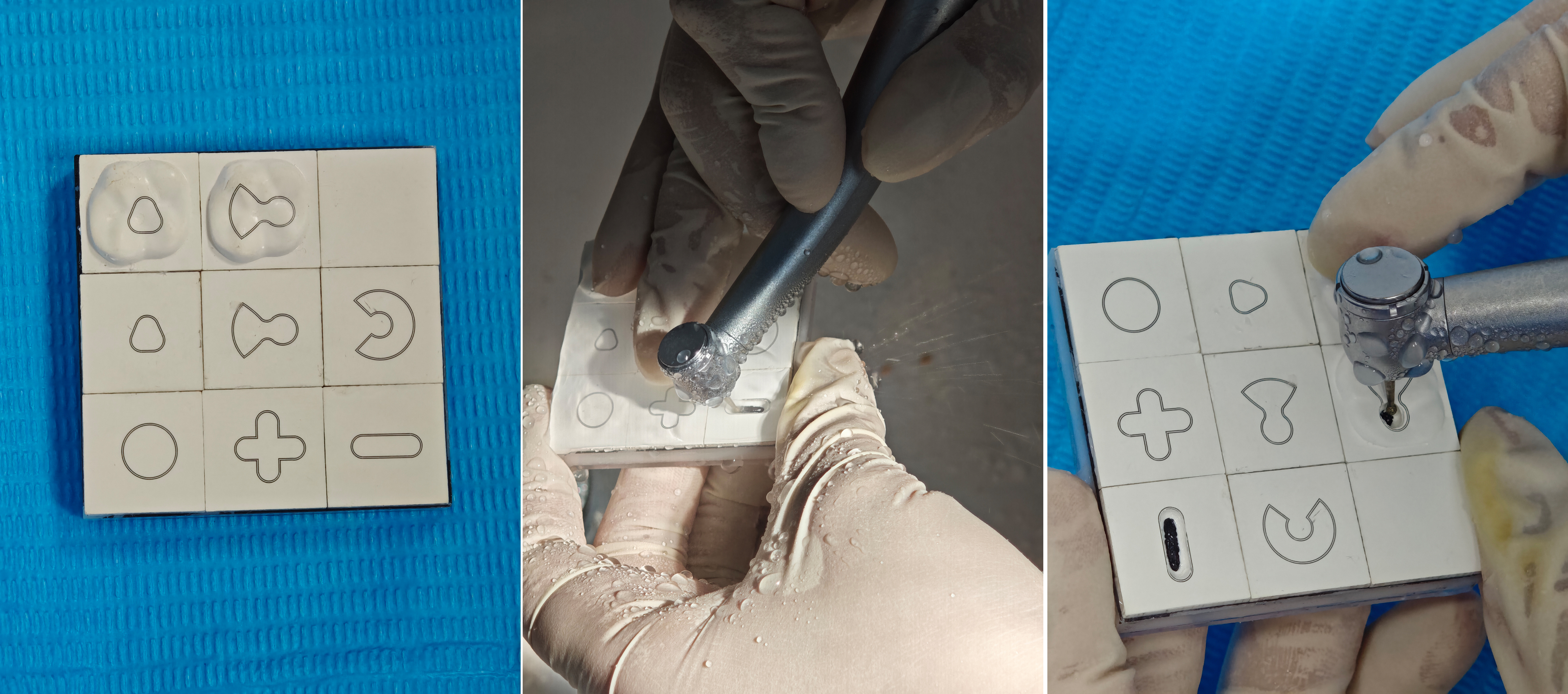

Supplement: Supplementary file 3 — Supplementary Material 3. [file 12909_2025_8525_MOESM3_ESM.jpg]
